# δ^13^C Determination of Benzoic Acid by High-Resolution Orbitrap MS: How Sample Introduction Strategy Governs Accuracy and Precision

**DOI:** 10.1021/jasms.6c00221

**Published:** 2026-09-11

**Authors:** Giovanni B. Bevilaqua, Hugo G. Machado, Júlio C. O. Ribeiro, Nerilson Marques Lima, Alexandre A. Ferreira, Ygor S. Rocha, Gabriel F. dos Santos, Boniek Gontijo

**Affiliations:** † Chemistry Institute, 67824 Federal University of Goiás, Goiânia 74690-900, Brazil; ‡ UNIFAL, Universidade Federal de Alfenas, Alfenas 37130-001, Minas Gerais, Brazil; § 42506 PETROBRAS Research and Development Center (CENPES), PETROBRAS, Rio de Janeiro 21941-915, Brazil

**Keywords:** isotope, ratio, δ13C, performed, mass, spectrometry, Orbitrap, benzoic, acid, HPLC, accuracy, σRSE, approximately, replicate, means, residuals, Exploris, analysis, injection

## Abstract

Stable carbon isotope ratio measurements (δ^13^C) are traditionally performed by isotope ratio mass spectrometry (IRMS), but recent advances in high-resolution Orbitrap mass spectrometry (Orbitrap MS) enable molecular-level isotope ratio analyses without conversion to simple gases. Here, we systematically evaluate and compare three sample-introduction strategies for Orbitrap-MS-based δ^13^C determination of benzoic acid—dual-inlet (DI), single-inlet (SI), and automated HPLC flow-injection (column-free mode)—assessing how each governs measurement accuracy and precision. Analyses were performed using paired injections of an isotopically certified benzoic acid reference (IAEA-601) and a benzoic acid sample previously characterized by elemental analysis–IRMS (EA-IRMS), following a seven-block bracketing sequence (Ref-Smp-Ref-Smp-Ref-Smp-Ref) to minimize temporal drift and to generate within-run triplicates. Across all configurations, high analytical precision was achieved, within-block relative standard error (σ_RSE_) consistently below approximately 0.6‰. Under DI conditions, replicate means closely approached the EA-IRMS benchmark (−27.77‰), with residuals of −0.40 to +0.50‰ on the Exploris 240 and −0.58 to −2.73‰ on the Q Exactive. SI analysis enabled the acquisition of a higher number of individual scans per block, contributing to lower σ_RSE_ (approximately 0.50–0.65‰) but exhibited larger accuracy offsets (residuals up to approximately 1–3‰), indicating greater susceptibility to systematic bias. Automated HPLC injection on the Exploris 240, optimized for concentration and AGC (50 μmol L^–1^ and 2.5 × 10^5^, respectively), delivered δ^13^C values of 28.1‰, −27.19‰, and −27.79‰ (residuals of approximately 0.20–0.45‰) with no significant differences among replicate means (ANOVA/Tukey, 95% confidence level). Collectively, these findings confirm Orbitrap-MS as a reliable alternative to IRMS for δ^13^C analysis of benzoic acid, and reveal that the choice of injection strategy is the primary determinant of the accuracy–precision–throughput balance achievable with this platform.

## Graphical abstract

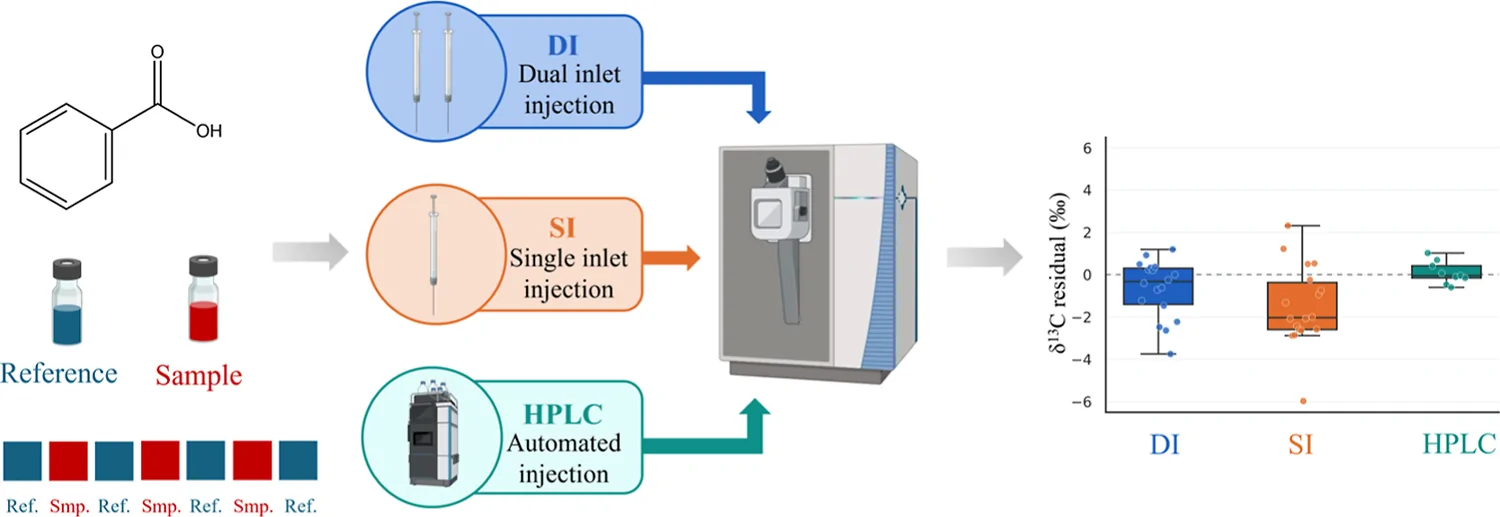

## 1 Introduction

The measurement of stable isotope ratios, particularly δ^13^C, has become a fundamental tool in chemical, environmental, and geochemical research, providing insights into reaction mechanisms,^1,2^ trace sources and fluxes of matter in ecosystems,^3,4^ forensic studies of food adulteration,^5^ doping control,^6^ and historical changes in environmental and petroleum systems.^7,8^ Traditionally, isotope ratio mass spectrometry (IRMS) has served as the benchmark technique due to its high precision and reproducibility across diverse sample types.^9,10^ However, IRMS requires complete conversion of analytes into simple gases (e.g., CO_2_, N_2_, H_2_), which inherently sacrifices intramolecular isotopic information, such as position-specific isotope ratios or clumped isotopologues, in favor of methodological simplicity and precision. Access to these detailed isotopic patterns could provide deeper mechanistic insights and broaden the scope of isotope-based applications.^11,12^

Recent advances in high-resolution mass spectrometry have introduced Orbitrap-MS as a promising alternative for stable isotope analyses.^13^ Electrospray ionization (ESI) coupled to an Orbitrap mass spectrometer enables direct molecular-level δ^13^C measurements without gas conversion, delivering high mass resolving power up to 1,000,000, sub-ppm mass accuracy, and a broad dynamic range.^13–15^ These attributes enable the discrimination of isotopologues and the quantification of rare isotopic species with precision approaching 1‰ or better, as demonstrated for compounds such as oxyanions,^16,17^ amino acids,^18,19^ hydrocarbons,^20^ polyfluoroalkyl substances (PFAS),^21,22^ pesticides,^23^ pharmaceuticals,^24^ and fatty acids.^25^

Nevertheless, isotope ratio measurements by Orbitrap-MS remain sensitive to experimental configurations, including ion optics, sample introduction, AGC target, ion population, injection time, and acquisition strategy.^26–29^ Dual-inlet (DI) approaches improve temporal stability by alternating sample and reference streams, reducing instrumental drift.^15,17^ Conversely, single-inlet (SI) systems offer operational simplicity even though they exhibit baseline fluctuations. More recently, automated HPLC injection methods have enabled high-throughput measurements with enhanced reproducibility and reduced risks of contamination.^25,30^ Comparative studies indicate that DI configurations consistently achieve superior long-term accuracy,^17,29^ whereas HPLC systems offer substantially greater sample throughput and improved operational robustness,^22,25,30^ underscoring their complementary rather than mutually exclusive strengths.

Within this context, benzoic acid serves as an ideal model compound for method development in isotope ratio analysis due to a unique combination of chemical and instrumental advantages. Structurally simple yet analytically relevant, it features a well-defined isotopic envelope and generates stable, high-intensity signals in negative-ion ESI, thereby enhancing mass resolution and minimizing background noise. Its low molecular weight and high solubility in polar solvents make it highly compatible with Orbitrap-MS workflows, whether in direct infusion or chromatographic modes. Moreover, benzoic acid is widely encountered in environmental matrices, petrochemical products, food preservatives, and biological systems, making it both technically convenient and scientifically meaningful as a benchmark analyte.^31–34^ Its inclusion in this study enables not only a precise assessment of Orbitrap-MS performance for δ^13^C measurements but also offers practical relevance for real-world sample applications.

Here, we evaluate how sample introduction strategy governs δ^13^C accuracy and precision in Orbitrap-MS measurements of benzoic acid, comparing three configurations—dual inlet (DI), single inlet (SI), and automated HPLC mode—across two Orbitrap platforms (Q-Exactive and Exploris 240). Against EA-IRMS reference values as the accuracy benchmark, we quantify within-block relative standard error (σ_RSE_), δ-residuals, and reproducibility for each configuration, thereby providing a performance map to guide analytical strategy selection for stable isotope applications in organic geochemistry and related fields.

## 2 Materials and Methods

### 2.1 Chemicals and Materials

Analytical-grade methanol (MeOH) was obtained from J.T. Baker. Benzoic acid (analytical grade) was purchased from Sigma-Aldrich, while its isotopically certified reference standard (IAEA-601; δ^13^C = −28.81‰) was supplied by the International Atomic Energy Agency (IAEA). Ammonium hydroxide (NH_4_OH, 28–30%), used as an additive to enhance negative-mode ionization, was purchased from Sigma-Aldrich. A list of benzoic acid isotopologues, relative abundances, and corresponding *m*/*z* values in negative mode is provided in Table 1.

**Table 1:** Isotopologues of Benzoic Acid^a^

mass range	*m*/*z*	abundance (%)	isotopolog
M0	121.028955	92.036729	C_7_H_5_O_2_
M + 1	122.032305	7.165648	C_6_^13^CH_5_O_2_
	122.033171	0.073806	C_7_H_5_O^17^O
	122.035232	0.069040	C_7_H_4_^2^HO_2_
M + 2	123.033200	0.369032	C_7_H_5_^18^OO
	123.035655	0.239106	C_5_^13^C_2_H_5_O_2_
	123.036521	0.005740	C_6_^13^CH_5_O^17^O
	123.037387	0.000015	C_7_H_5_^17^O_2_
	123.038582	0.005390	C_6_^13^CH_4_^2^HO_2_
	123.039448	0.000060	C_7_H_4_^2^H^17^OO
	123.041509	0.000020	C_7_H_3_^2^H_2_O_2_

a *These calculations were based on IsotoPy software available on: https://www.isotopy.com.br/.

### 2.2 Sample Preparation

Stock solutions (1 mmol L^–1^) of the benzoic acid sample (analytical grade) and the reference standard (isotopically certified reference standard) were prepared in methanol containing 1% NH_4_OH to enhance deprotonation efficiency. Working dilutions at concentrations of 0.5, 1.0, 5.0, 10.0, 25.0, and 50.0 μmol L^–1^ were freshly prepared and filtered through 0.22 μm PTFE filters prior to Orbitrap mass spectrometry analysis.

### 2.3 Evaluation of Carbon Isotope Content (δ^13^C) by Isotope Ratio Mass Spectrometry

The expected isotopic composition of the benzoic acid sample was determined using the conventional elemental analyzer coupled to an isotope ratio mass spectrometer (EA-IRMS) Flash EA IsoLink CN IRMS System (Thermo Scientific). The stable carbon isotope content (δ^13^C) was quantified by converting the samples to CO_2_ gas, allowing precise measurement of the ^13^C/^12^C isotope abundance ratio (‰) relative to the VPDB standard.

Solid samples (150 μg) were sealed in tin capsules and introduced into the elemental analyzer, where they underwent flash combustion in a reactor (above 1000 °C) under an oxygen atmosphere. The results are expressed in delta notation (δ^13^C, ‰), representing the relative deviation between the isotopic ratio of the sample and that of the international VPDB (Vienna Pee Dee Belemnite) standard. The analysis was performed in duplicate. The primary reference material used was NBS-22, and a crude oil sample served as an in-house working standard.

### 2.4 Instrumentation

Stable carbon isotope analyses were performed on two high-resolution Orbitrap mass spectrometers, a Thermo Scientific Q-Exactive and a Thermo Scientific Exploris 240, both equipped with heated electrospray ionization (H-ESI) sources and operating in negative ionization mode. The Q-Exactive system was used for dual-inlet (DI) and single-inlet (SI) infusion experiments, whereas the Exploris 240 supported all three injection modes, including automated HPLC-based flow injection. The Q-Exactive platform did not accommodate HPLC-based auto-injection due to physical and operational incompatibilities with its ion source configuration.

Instrumental parameters were optimized for isotopic resolution and signal stability. Although both instruments were operated under comparable conditions, certain parameters, such as ion optics voltages, AGC target, and injection time, differed slightly between systems due to inherent differences in hardware design and dynamic range characteristics. Details of the principal operating parameters for each instrument are provided in Table S1 (Supporting Information).

### 2.5 Injection Systems and Acquisition Strategies

Before comparing the different injection strategies, two key instrumental parameters (analyte concentration and automatic gain control (AGC)) were optimized to ensure stable ion statistics and reproducible isotope-ratio measurements. These optimizations were performed on the Orbitrap Exploris 240 using the automated HPLC flow-injection system, chosen for its practicality in handling multiple solutions. Benzoic acid reference and sample solutions were analyzed independently (i.e., without alternating injections) in triplicate 15 min acquisitions. Each acquisition included an initial 1 min delay volume and a 6 min flushing period, resulting in an effective analysis window of 8 min (between 1 and 9 min). Concentrations ranging from 1–50μmol L^–1^ and AGC targets of 5 × 10^4^, 1.5 × 10^5^, and 2.5 × 10^5^ were evaluated. These preliminary experiments focused exclusively on assessing precision and signal-stability metrics (more details are provided in the Supporting Information, Tables S2 and S3). The optimized conditions were then applied in all subsequent isotope-ratio analyses.

Three distinct sample introduction systems were employed (dual-inlet (DI), single-inlet (SI), and automated HPLC flow injection), each evaluated for precision and accuracy in δ^13^C determination. Across all configurations, analyses were performed using paired injections of benzoic acid reference standard IAEA-601 (with certified δ^13^C value) and a benzoic acid sample previously characterized by EA-IRMS.

All measurements followed a seven-block bracketing sequence, alternating injections of reference and sample according to the pattern (Ref-Smp-Ref-Smp-Ref-Smp-Ref). This scheme produces three δ^13^C values, corresponding to within-run triplicates, with each sample block bracketed by two reference blocks. The alternating design effectively minimizes temporal drift and short-term instabilities typical of electrospray ionization-based isotope ratio measurements, thereby enhancing reproducibility.^15^

- Dual-Inlet (DI) Configuration: DI experiments were performed following the protocol of dos Santos et al. (2025),^25^ using a 6-port divert valve to alternate sample and reference flows in real time. This configuration enables a complete seven-block sequence within a single continuous acquisition, allowing simultaneous monitoring of both streams while minimizing drift. Flow rates were maintained at 5 μL min^–1^ using Chemyx syringe pumps fitted with 500 μL Hamilton syringes, and all solutions were prepared in LC-MS-grade methanol. Experiments were performed in triplicate on both Orbitrap instruments.
- Single-Inlet (SI) Configuration: In SI mode, either the sample or the reference solution was infused into the mass spectrometer via a Chemyx syringe pump at 5 μL min^–1^. Each acquisition lasted 35 min, after which the syringe and tubing were thoroughly rinsed with 2.5 mL LC–MS-grade methanol to prevent carryover. The SI configuration required separate injections for each block of the seven-block sequence, while maintaining the same temporal alternation as in the DI setup. The analyses were performed in triplicate on both Orbitrap instruments.
- Automated HPLC Injection: automated injections were carried out on a Vanquish Neo microflow system coupled to the Orbitrap Exploris 240. The HPLC was operated without a chromatographic column, serving as an autosampler for direct flow injection analysis (FIA). Benzoic acid solutions prepared using LC–MS-grade methanol were delivered at 5 μL min^–1^, with a 100 μL injection loop, from which 50 μL were injected per cycle to generate a broad plateau signal (∼10 min in width). The total analysis time was 15 min, including a flushing period to eliminate carryover. Each injection represented one block within the seven-block sequence, and the entire protocol was repeated in three independent triplicate runs, consistent with the DI and SI experiments.

Across all configurations and instruments, the seven-block bracketing design produced three δ^13^C values, corresponding to within-run triplicates. Repeating the full protocol in three independent runs provided a total of nine δ^13^C determinations per condition (i.e., 3 × 3 replicates), which were subsequently evaluated by one-way ANOVA followed by Tukey’s post-hoc tests to assess intra and inter-run reproducibility.

### 2.6 Data Processing

All raw data were processed using IsotoPy, an in-house software platform developed by our research group at the Federal University of Goiás available in https://isotopy.com.br/. The software, written in Python 3, integrates the Thermo Fisher Scientific RawFileReader library (C#/.NET) to enable high-performance access to proprietary .RAW files and direct extraction of Orbitrap spectral data.^25^

The program automates the complete workflow of Orbitrap-based isotope ratio analysis, encompassing signal extraction, scan-level filtering, noise evaluation, ion-count computation, isotopic ratio determination, and δ-value calculation. It was designed to reproduce and extend the quantification framework implemented in Thermo Fisher Scientific’s IsoX software. The ion count (IC) for each isotopologue is computed according to a model derived from ion-statistics theory, expressed as

1 $$IC=\frac{S}{N}\times 3\times \sqrt{\frac{R_{n}}{r}}\times \sqrt{\mu }$$

where *S* is the signal intensity, *N* the baseline noise, *r* the experimental resolution, μ the number of microscans, and *R*_*N*_ = 240,000 is the reference resolution at which the empirical constant 3 was determined.^15^

The isotopic ratio ^13^R is defined as the ratio of IC for the heavy (^13^C) and light (^12^C) isotopologues

2 $$13R=\frac{{IC}_{13C}}{{IC}_{12C}}=\frac{S_{13C}/N_{13C}}{S_{12C}/N_{12C}}$$

Since both isotopologues are recorded under identical instrumental conditions, all constant factors cancel, and the final ratio depends solely on relative signal-to-noise behavior.

Prior to the ratio calculation, IsotoPy performs a series of preprocessing steps that vary slightly depending on the injection method. In DI analyses, the initial minute of each block, which corresponds to valve switching, is automatically excluded to remove transient artifacts. In Supporting Information analyses, where each block corresponds to a separate syringe infusion, data were trimmed from minute 2 to minute 32 for a 35 min run. In automated HPLC flow-injection analyses, only the plateau region of the total-ion chromatogram, from minute 1 to minute 9 for a 15 min run, is retained.

All retained scans are subjected to outlier detection using the Median Absolute Deviation (MAD) score method with a 2-MAD threshold in a sliding-window implementation. Scans lacking one of the isotopologue peak (“zero scans”) are discarded to prevent artificial bias.

After filtering, IsotoPy computes isotopic ratios on a scan-by-scan basis, averages them within each block, and propagates uncertainties from ion statistics. The output includes acquisition-level metrics such as ion count, isotopic ratio, standard error of the mean (SEM), within-block relative standard error (σ_RSE_, the relative standard error-RSE × 1000-in ‰), and shot-noise estimates (σ_SN_). More details in Supporting Information.

### 2.7 Calculation of δ-Values

The isotopic delta (δ) values express the deviation of the sample’s isotopic composition from that of a reference standard, calculated as

3 $$\delta_{sample/s\tan dard}=\left(\frac{R_{smp}}{R_{std}}-1\right)\times 1000$$

where *R* represents the isotopic ratio (^13^C/^12^C).

In all experiments, the seven-block bracketing scheme (described in Section 2.4) was applied. δ^13^C values were computed using the standardization approach,^29^ in which a linear regression is fitted to the average isotope ratios of the four reference blocks (1, 3, 5, and 7). The regression predicts the expected reference ratio for each sample block (2, 4, and 6), thereby compensating for potential instrumental drift. The measured sample ratios are then compared with these predicted values through eq 3.

From this procedure, IsotoPy reports two precision parameters associated with δ-value determination.

- Propagated relative standard error (σ_pRSE_), which combines the within-block relative standard error (σ_RSE_) with the prediction uncertainty of the regression model used for drift correction.
- Experimental reproducibility error (σ_ER_), which is the standard deviation among the three δ^13^C values derived from a single seven-block sequence, reflecting the overall reproducibility of the measurement.

The final δ^13^C for each run corresponds to the mean of the three individual δ-values obtained from the model-based correction, with σ_pRSE_ and σ_ER_ providing complementary assessments of propagated and experimental precision, respectively. Further mathematical definitions and implementation details for these parameters are provided in Supporting Information.

δ-Values were subsequently adjusted to the VPDB scale using the certified δ^13^C value of the benzoic acid standard (IAEA-601), according to

4 $${\delta^{13}C}_{smp/VPDB}={\delta^{13}C}_{smp/std}+{\delta^{13}C}_{std/VPDB}+\frac{{\delta^{13}C}_{smp/std}\times {\delta^{13}C}_{std/VPDB}}{1000}$$

where ${\delta^{13}C}_{std/VPDB}$ corresponds to the certified value of IAEA-601 (−28.81‰). The benzoic acid sample used in this study had a δ^13^C value of −27.77‰ vs VPDB, as independently determined by EA-IRMS, and this value was adopted as the accuracy reference throughout this work.

The resulting dataset, therefore, includes block-level δ^13^C values, within-run averages, and their associated uncertainties, enabling quantitative comparison of precision, reproducibility, and accuracy across injection systems and instruments.

## 3 Results and Discussion

To assess the analytical performance of Orbitrap-based isotope ratio measurements, benzoic acid was selected as a model compound. This molecule provides a clean, well-resolved isotopic envelope within the *m*/*z* 120–124 range, allowing precise evaluation of instrumental behavior and isotope-ratio stability (Figure 1). This selection is consistent with previous Orbitrap-based isotope studies on small molecules, including oxyanions,^16,17^ acetate,^29^ and amino acids.^18,19,35^ A representative high-resolution mass spectrum shows the isotopic distribution of benzoic acid acquired in selected ion monitoring (SIM) mode. The monoisotopic (^12^C_7_H_6_O_2_^–^, *m*/*z* 121.0296) and ^13^C-substituted (^13^C_1_^12^C_6_H_6_O_2_^–^, *m*/*z* 122.0328) peak fully baseline-separated at a resolving power of 60,000 (FWHM at *m*/*z* 200), demonstrating that even moderate-resolution Orbitrap settings are sufficient for accurate isotopologue discrimination without spectral overlap or interferences. This isotopic pattern served as the analytical basis for all subsequent precision and accuracy evaluations.

**Figure 1:**
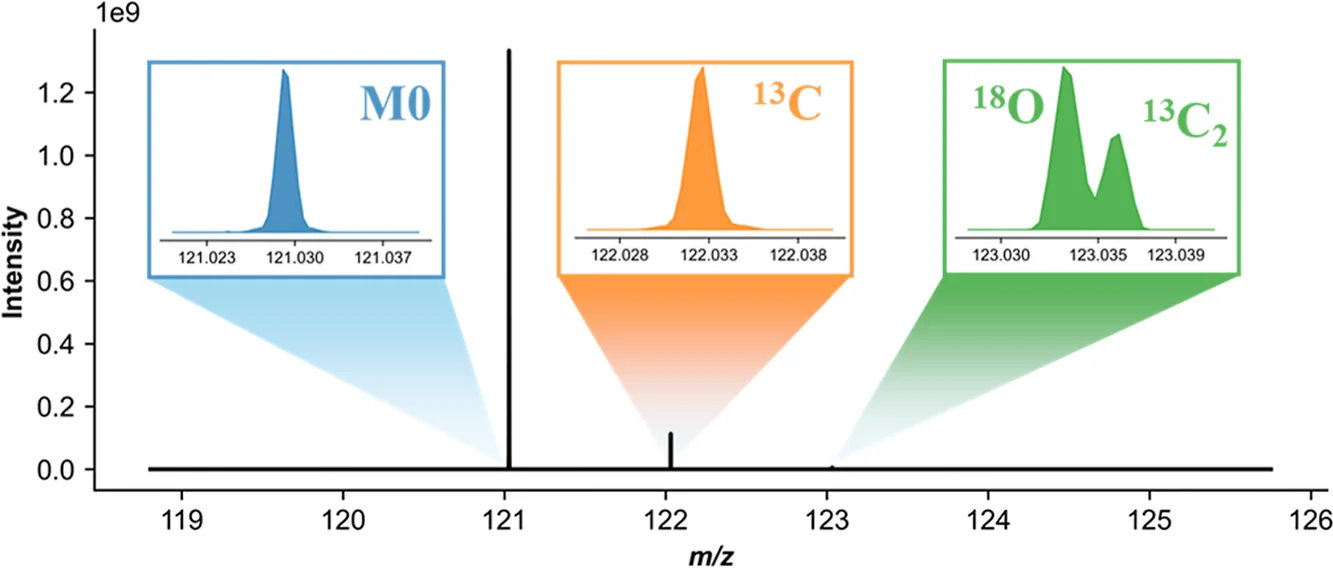
SIM spectrum of benzoic acid over the *m*/*z* 120–124 range, acquired for Orbitrap-MS–based isotopic analysis. The M0, M+1, and M+2 isotopologues are depicted, each distinctly separated owing to the exceptional mass resolving capability of the Orbitrap instrument.

Prior to testing the different injection strategies (DI, Supporting Information, and automated HPLC), two critical parameters (the AGC target and analyte concentration) were optimized. These optimizations were conducted using the Orbitrap Exploris 240 coupled to the automated HPLC flow-injection system, chosen for its convenience in handling multiple solutions. The goal was to ensure that all subsequent comparative experiments would be performed under uniform, optimized conditions.

### 3.1 Optimization of Analytical Conditions

#### 3.1.1 Analyte Concentration

The effect of analyte concentration on the measured ^13^C/^12^C ratio was evaluated over the 1–50 μmol L^–1^ range for both the benzoic acid sample and reference. As shown in Figure 2a,b, the isotope ratio increased progressively with concentration, gradually approaching a stable plateau above 10 μmol L^–1^. At the lowest concentrations (1 and 5 μmol L^–1^), the ^13^R values were systematically lower for both reference and sample, indicating bias associated with low ion populations. The corresponding precision metrics (σ_RSE_ and σ_RSE_/σ_SN_) showed no substantial variation across the tested concentrations (Table S3).

**Figure 2:**
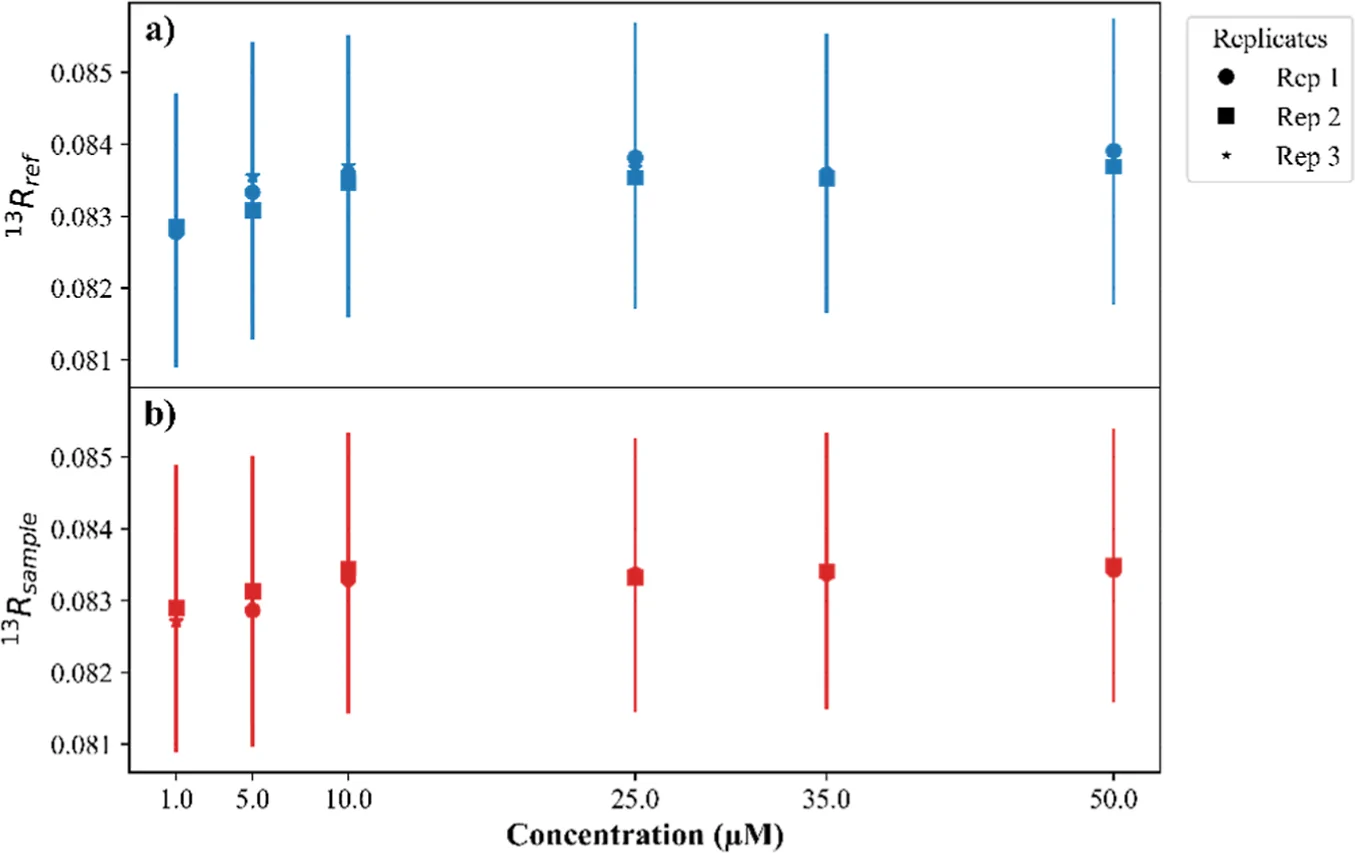
Effect of analyte concentration (1–50 μmol L^–1^) on ^13^R of benzoic acid using HPLC-based injection on the Orbitrap Exploris 240.

Visual inspection of Figure 2 reveals that the isotope ratio remained stable between 10 and 50 μmol L^–1^, indicating a concentration plateau over which both accuracy and precision are maintained. We therefore selected 50 μmol L^–1^ as the working concentration for all subsequent experiments. This choice ensures stronger ion signals and avoids potential blank effects that can occur at low concentrations, when ion populations are insufficient to maintain consistent ion packet formation. Under such conditions, the instrument may reach its maximum injection time and operate outside the AGC control regime, allowing transient fluctuations in the total ion current (TIC) to dominate. This instability can distort the isotopic ratio and introduce systematic bias in δ-value determinations.

As an illustration of this concentration-dependent bias, the δ-values derived from the 1 μmol L^–1^ acquisition deviated markedly from those obtained at the plateau region (10–50 μmol L^–1^). The mean offset was approximately 6.8‰ for the sample and 9.9‰ for the reference, confirming that measurements performed at very low concentrations may compromise isotopic accuracy. By adopting concentration within the plateau region (where the ^13^R ratio remains constant), we minimize these biases and ensure stable AGC-controlled acquisitions. Notably, concentrations in the 25–50 μmol L^–1^ range are also consistent with values commonly employed in previous Orbitrap-based isotope-ratio studies of structurally similar compounds.^17,30^

#### 3.1.2 Automatic Gain Control (AGC)

The AGC target determines the number of ions accumulated per scan and thus directly affects the achievable precision in isotope-ratio analyses. Three AGC settings were evaluated: 5 × 10^4^ (5%), 1.5 × 10^5^ (15%), and 2.5 × 10^5^ (25%). Increasing AGC improved precision systematically: σ_RSE_ decreased from ∼0.78‰ at 5 × 10^4^ to ∼0.39‰ at 2.5 × 10^5^ (Figure 3, for more details see Table S2). The σ_RSE_/σ_SN_ also approached unity under the highest AGC condition, indicating that the measurement precision was limited primarily by ion-counting statistics.

**Figure 3:**
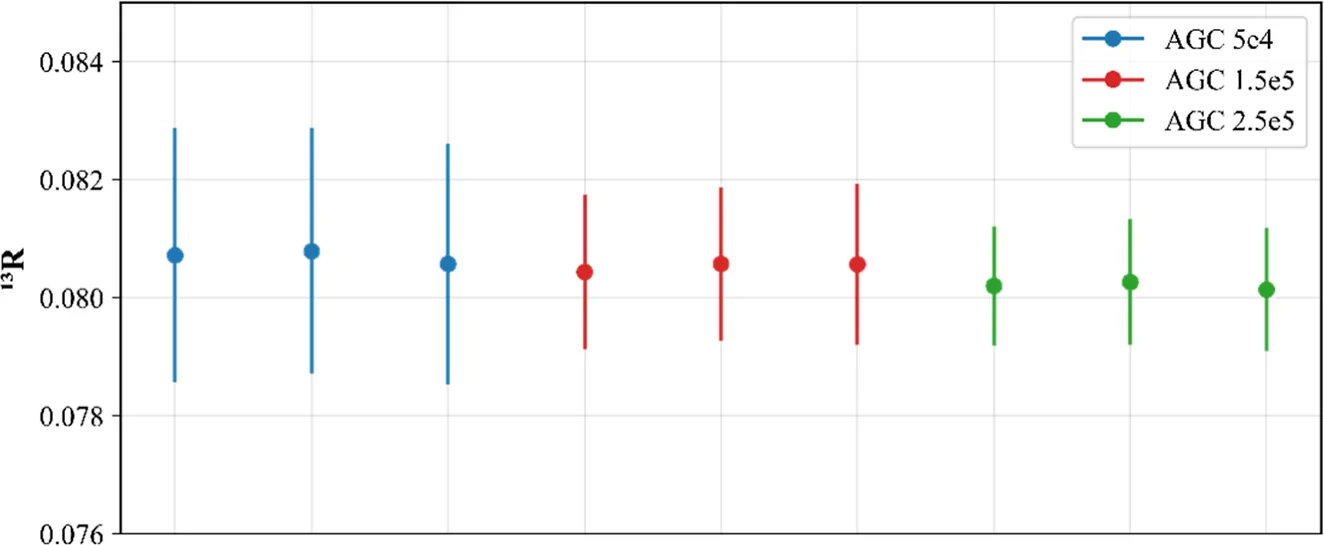
Effect of automatic gain control (AGC) target on ^13^R of benzoic acid using HPLC-based injection on the Orbitrap Exploris 240. Three AGC settings were tested (5 × 10^4^, 1.5 × 10^5^, and 2.5 × 10^5^).

Within the evaluated range (5 × 10^4^ to 2.5 × 10^5^), the highest AGC target produced the lowest σ_RSE_ for benzoic acid under the present HPLC flow-injection conditions. Therefore, 2.5 × 10^5^ should be interpreted as the best setting among those tested, rather than as a universal AGC optimum. Previous Orbitrap isotope-ratio protocols have used higher AGC targets under different experimental conditions. For example, Kantnerová et al. (2024) used a normalized AGC target of 3 × 10^5^ for flow-injection analysis and 1 × 10^6^ for direct-infusion measurements, while emphasizing that AGC values must be adapted to the analyte, instrument, and ionization conditions and evaluated together with TIC × IT and possible overfilling effects.^15^

In addition, Machado et al. (2026) showed that Orbitrap isotope-ratio precision is governed by an intermediate signal-stability plateau: low ion populations increase statistical noise, whereas excessive ion populations or co-accumulated ions may introduce nonlinear and space-charge-related biases. They also demonstrated that reaching the maximum injection time can prevent the instrument from attaining the programmed AGC target, resulting in subtarget ion populations and degraded precision.^27^ Future experiments should extend the AGC range and evaluate injection-time distributions, TIC × IT, effective ion counts, precision, and isotope-ratio accuracy. The selected working conditions (50 μmol L^–1^ and AGC = 2.5 × 10^5^ the highest target tested) were subsequently applied across all injection configurations and instruments.

### 3.2 Evaluation of Injection Strategies

With the analytical conditions optimized, three injection strategies were systematically evaluated to compare their precision, reproducibility, and accuracy in δ^13^C determination: DI, Supporting Information, and automated HPLC flow injection. Across all setups, δ^13^C values were computed relative to the certified benzoic acid standard IAEA-601 (δ^13^C = −28.81‰ VPDB), while the Sigma standard sample (δ^13^C = −27.77‰ VPDB by EA-IRMS) served as the accuracy benchmark. Each experiment followed a seven-block alternating sequence, yielding three δ^13^C values per run (within-run triplicates) and three independent runs per configuration (3 × 3 replicates).

#### 3.2.1 Dual Inlet Experiments

Figure 4a,b summarize the DI results for Orbitrap instruments, acquired under identical optimized conditions for this method (50 μmol L^–1^; AGC = 2.5 × 10^5^). Panel (a) shows the δ^13^C values derived from each individual sample block (three per replicate; nine per instrument), while panel (b) presents the mean δ^13^C obtained from each complete seven-block sequence (three replicate per instrument).

**Figure 4:**
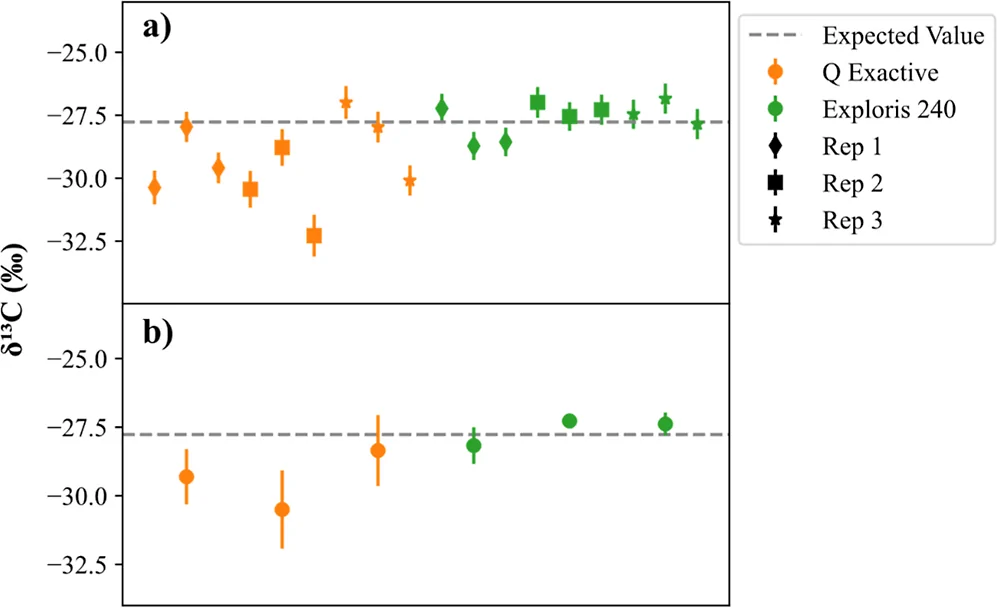
δ^13^C (‰, VPDB) values for benzoic acid obtained using the dual-inlet injection method on two Orbitrap platforms: Q Exactive (orange) and Exploris 240 (green). (a) Individual technical replicates; (b) replicate-set means (Rep 1–3), with symbols indicating each replicate set. Error bars represent 1σ (standard deviation). The dashed line denotes the expected δ^13^C value (−27.77‰).

The Exploris 240 exhibits markedly lower dispersion both within and between blocks compared with the Q-Exactive (Figure 4a). This translates into systematically lower σ_RSE_, σ_pRSE_ values and σ_RSE_/σ_SN_ ratios closer to unity (see Table S4), indicating performance approaching the ion-counting (shot-noise) limit. In contrast, the Q-Exactive displays broader δ-value distributions, with larger propagated σ_RSE_ and more variable block-to-block behavior. Practically, this means the Exploris maintains more uniform packet formation over time, while the Q-Exactive shows greater stochastic variability in its scan-to-scan statistics. Consequently, the Exploris delivers superior short-term precision, which is the critical metric for assessing the intrinsic stability of Orbitrap-based isotope ratio measurements.

Final δ^13^C values averaged per replicate (Figure 4b) reveal that both instruments converge toward the IRMS benchmark (−27.77‰), but with different degrees of proximity. Exploris 240 yielded residuals of −0.40, +0.50 and +0.39‰, whereas Q-Exactive produced −1.54, −2.73, and −0.58‰. Thus, both systems achieved acceptable accuracy, but Exploris exhibited smaller absolute deviations and tighter clustering around the true value.

Within each instrument, one-way analysis of variance (ANOVA) followed by Tukey’s post hoc test was applied to assess the precision of this analytical mode. As summarized in Table S5, the results confirm the method’s reproducibility, with no statistically significant differences at the 95% confidence level.

#### 3.2.2 Single Inlet

To overcome one of the primary limitations of the DI configuration, specifically, the restricted number of scans per run imposed by the limited syringe volume, we implemented an Supporting Information strategy. Figure 5a,b summarize the δ^13^C results obtained using the Supporting Information configuration on the Q-Exactive and Exploris 240 platforms, showing the δ^13^C values derived from each individual sample block, and the mean δ^13^C obtained. In this approach, each block of the seven-block sequence was acquired as an independent 35 min infusion, which increases the number of scans collected per block relative to the DI workflow and therefore provides a direct means to evaluate within-block stability and replicate-level performance under extended acquisition conditions.

**Figure 5:**
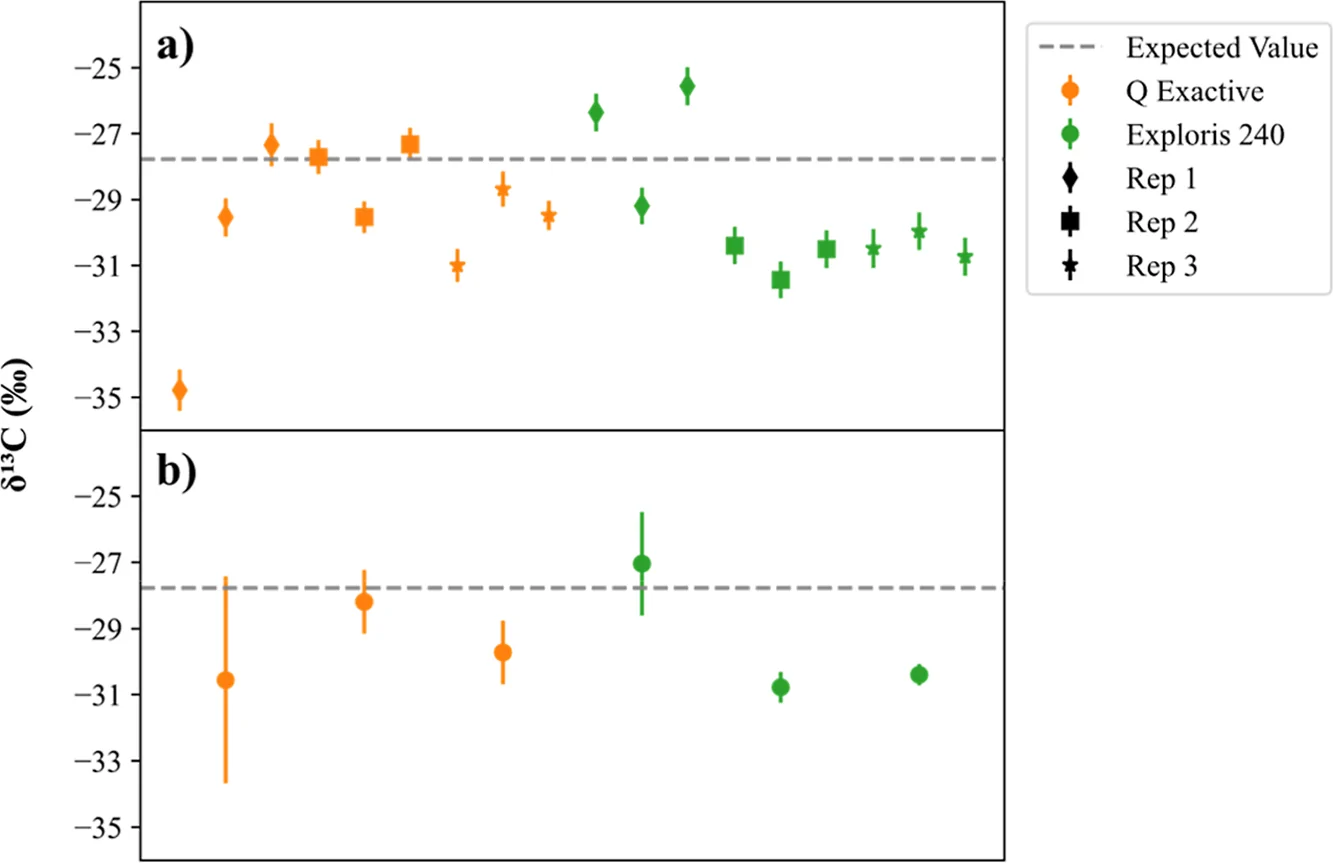
δ^13^C (‰, VPDB) values for benzoic acid obtained using the single-inlet injection method on two Orbitrap platforms: Q exactive (orange) and Exploris 240 (green). (a) Individual technical replicates; (b) replicate-set means (Rep 1–3), with symbols indicating each replicate set. Error bars represent 1σ (standard deviation). The dashed line denotes the expected δ^13^C value (−27.77‰).

Supporting Information measurements yielded consistently low σ_RSE_ across both platforms (σ_RSE_ typically ∼0.50–0.65‰ on the Q-Exactive and ∼0.56–0.59‰ on the Exploris 240). The longer acquisition blocks in the Supporting Information configuration enabled the collection of a higher number of individual scans per block, thereby reducing the standard error of the block mean and improving short-term precision. However, the extent to which measurements approached the shot-noise floor differed by platform: the Exploris 240 operated closer to the ion-statistics limit (σ_RSE_/σ_SN_ ≈ 1.39–1.41), whereas the Q-Exactive exhibited σ_RSE_ values that remained ∼2.5–2.9 times σ_SN_, indicating additional contributions beyond counting statistics despite improved short-term stability.

Supporting Information determinations showed larger deviations from the EA-IRMS benchmark (−27.77‰) than those observed under DI conditions. On the Q-Exactive, replicate means spanned −30.55‰ to −28.19‰, corresponding to residuals of −2.78‰ to −0.42‰ compared to the IRMS expected value. On the Exploris 240, replicate means ranged from −30.77‰ to −27.04‰, with residuals between −3.00‰ and +0.73‰. These results indicate that, although Supporting Information can improve within-block precision, δ^13^C accuracy in this configuration remains more susceptible to systematic bias, plausibly linked to concentration and/or equilibration effects during independent block acquisitions, as discussed for Supporting Information measurements.

Statistical evaluation of replicate means was performed within each instrument using one-way ANOVA followed by Tukey’s post hoc test (95% confidence level; Table S5). For the Q-Exactive, ANOVA did not detect significant differences among replicates (*p* = 0.5528), and all Tukey pairwise comparisons were non-significant (no rejections), supporting within-set reproducibility under Supporting Information operation. In contrast, the Exploris 240 exhibited a significant replicate effect by ANOVA (*p* = 0.0156), and Tukey’s test indicated significant differences between REP-1 vs REP-2 and REP-1 vs REP-3 (rejections = True), corroborating reduced reproducibility for the Supporting Information dataset on this platform, consistent with the observed instability in the early injection blocks.

#### 3.2.3 HPLC Injection

As an alternative to the other injection strategies, automated HPLC-based injections were employed, a methodology previously adopted in other studies^15,17,22,25,30^ to streamline workflows and reduce laboratory costs. In the present study, this approach was evaluated exclusively on the Orbitrap Exploris 240, using experimental conditions distinct from those applied to the other injection modes, given its inherently higher level of automation.

For this method, the 15 min runs generated the total ion chromatogram (TIC) shown in Figure 6. The stable signal interval between 1 and 9 min was selected based on the TIC profile. Using IsotoPy, scans acquired within this interval were retained for isotope-ratio analysis, whereas scans outside this interval were excluded. The scan-level ^13^C/^12^C ratios calculated from the retained scans yielded a mean within-block σ_RSE_ of 0.40‰ across three independent HPLC injections, with no systematic temporal trend in the isotope ratio. The final δ^13^C determinations showed a standard deviation of 0.37‰. These results distinguish the precision achieved within individual acquisition blocks from the repeatability obtained across independent injections.

**Figure 6:**
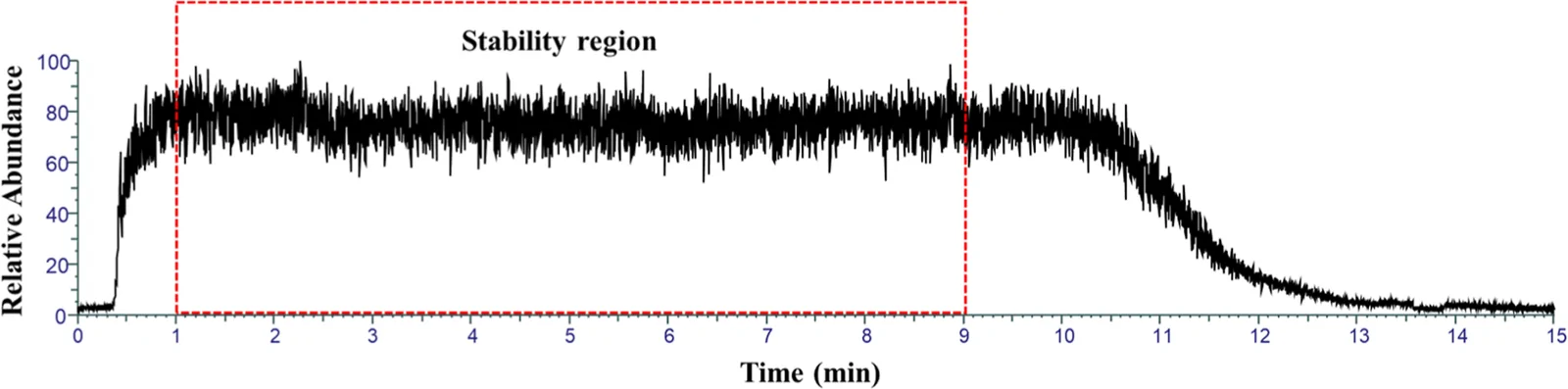
Total ion chromatogram (TIC) obtained for benzoic acid during a 15 min LC run with a 50 μL injection. The highlighted region (1–9 min, red box) corresponds to the stable plateau of the infusion profile, from which scans were extracted for δ^13^C analysis. This trimming removed non-steady-state signals associated with the injection front and elution tail, ensuring stable ion statistics for isotope ratio determination.

The precision achieved in the present study is consistent with the ion-counting framework described by Kantnerová et al. (2024) according to which the relative standard error decreases as the number of accumulated ions increases.^15^ Longer acquisitions have resulted in lower measurement uncertainties, including a σ_RSE_ of 0.07‰ for ^13^C/^12^C during a 24 h acetone measurement and a σ_pRSE_ of 0.4‰ for ^13^C/^12^C using 60 min methionine measurements.^28^ In comparison, the 8 min window employed here represents a practical compromise among isotope-ratio precision, sample consumption, analytical throughput, and the degree of automation provided by the HPLC-based sample-introduction approach.

HPLC injection mode was established after optimizing both analyte concentration and AGC for this specific workflow. The selected conditions (50 μmol L^–1^; AGC target = 2.5 × 10^5^) provided stable ion statistics and consistently low uncertainty estimates, with σ_RSE_ and σ_pRSE_ (Figure S5) lower than those observed for the dual-inlet configuration under our experimental conditions, supporting the improved short-term stability expected from automated sample introduction.

Under the optimized conditions for this method, the replicate δ^13^C values were −28.1‰, −27.19‰, and −27.79‰ (Figure 7). This performance is consistent with the expectation that automated injections reduce variability sources inherent to syringe handling, manual cleaning, and flow switching, thereby improving overall measurement robustness.

**Figure 7:**
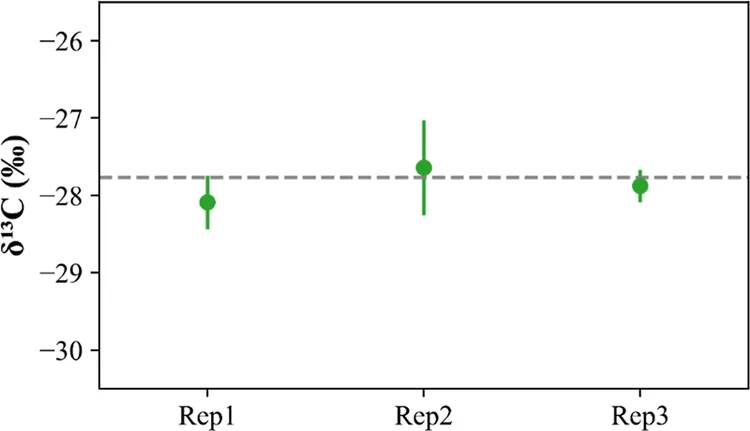
Analysis of benzoic acid using the HPLC injection configuration by Orbitrap Exploris 240. δ^13^C values for benzoic acid obtained across three independent determinations (green symbols); error bars represent the variability associated with each measurement (as defined in the Methods). The horizontal dashed line denotes the EA-IRMS δ^13^C value (VPDB).

To place the precision achieved in the present study in context, it is important to distinguish within-block precision from repeatability across independent determinations. Under the optimized HPLC-injection conditions, the mean within-block σ_RSE_ was 0.40‰, whereas the standard deviation among three independent δ^13^C determinations was 0.37‰ (1σ, *n* = 3). For the Supporting Information measurements, σ_RSE_ values were typically 0.50–0.65‰ on the Q Exactive and 0.56–0.59‰ on the Exploris 240. This performance is within the sub-permil range reported for Orbitrap-based isotope-ratio measurements. For example, Nelson et al. reported measurement precisions of 0.7‰ for intact PFOA and 0.2‰ for its decarboxylated fragment,^21^ whereas Wojtal et al.,^22^ using microflow HPLC-ESI coupled to an Orbitrap Exploris 240, reported errors of approximately 0.8–1.5‰ for PFOA measurements. Direct numerical comparison should nevertheless be interpreted cautiously because these studies differed in analyte identity, ionization conditions, acquisition duration, ion population, calibration strategy, and definition of the precision metric. Therefore, the precision obtained here represents a practical compromise between sub-permil precision, an approximately 8 min acquisition window, automation, and sample throughput.

Finally, within-set reproducibility across the three independent runs was evaluated using one-way ANOVA followed by Tukey’s post hoc test (Table S5). No statistically significant differences were observed among replicate means at the 95% confidence level (ANOVA *p* = 0.6029; all Tukey pairwise comparisons non-significant), confirming that the HPLC injection mode provides reproducible δ^13^C determinations under the proposed protocol.

#### 3.2.4 Comparison of Sample Injection Methods

The three injection strategies evaluated-dual inlet (DI), single inlet (SI), and automated HPLC injection - exhibited distinct strengths and limitations (Table 2). DI provided good accuracy, with σ_RSE_ values consistently tracking the shot-noise limit and residuals typically below 0.5‰, owing to continuous bracketing of reference and sample.^13,17,29^ In contrast, the Supporting Information configuration, which uses a single syringe alternately filled with standard or sample, enabled extended acquisition blocks and, consequently, the collection of a higher number of individual scans per block. This increased number of scan-level isotope-ratio measurements contributed to lower σ_RSE_. However, accuracy was more strongly affected, with residuals of ∼1–3‰ reported in both our study and by Mueller et al. (2024). Automated HPLC injection combined the advantages of stability and ease of operation, eliminating manual syringe handling. This method delivered reproducibility comparable to DI, but with improved accuracy and operational efficiency, as also demonstrated for fatty acids and complex lipids.^22^ Overall, our results confirm that while DI remains a classic method with reproducibility and accuracy, Supporting Information offers simplicity and but requires further investigation and HPLC provides a robust, automated alternative, particularly suited for complex or high-throughput applications.

**Table 2:** Comparison of Injection Strategies for Orbitrap-Based Isotope Ratio Mass Spectrometry (IRMS)^a^

injection strategy	configuration	advantages	limitations	representative studies
dual inlet (DI)	two syringes (one for reference, one for sample), alternating via valve	minimizes instrumental drift; direct bracketing of reference and sample; high long-term accuracy	limited scan number per block due to syringe volume; requires thorough cleaning; more complex hardware	Eiler 2017; Hilkert 2021; Csernica 2023; present study
single inlet (SI)	one syringe alternately filled with reference or sample	simpler setup; extended acquisition blocks with a higher number of individual scans; reduced σ_RSE_ approaching shot-noise limit	manual switching/cleaning; potential carry-over; accuracy sensitive	Csernica 2023; mueller 2024; present study
HPLC injection	autosampler delivers sample and reference via 50–100 μL loop; no chromatographic separation	automation; high reproducibility; reduced carry-over; scalable to high-throughput workflows	-	dos santos 2025; Hilkert 2021; Machado 2026; Kantnerová 2024; present study

a The table summarizes the main configurations, advantages, limitations, and representative studies for dual-inlet (DI), single-inlet (SI), and automated HPLC.

A limitation of the present study is the use of single-point isotope referencing. Although this approach has been successfully applied in recent Orbitrap-based isotope-ratio studies, it does not independently constrain the slope of the instrumental isotope-response function and therefore cannot exclude scale compression or expansion. Recent investigations have demonstrated context-dependent behavior: Bernet et al. (2025) reported near-unity slopes and superior precision using one-point referencing compared with two-point calibration,^36^ whereas Canavan et al. (2026) showed that two-point calibration can compensate for instrument-specific scale expansion.^24^ Because the IAEA-601 reference (−28.81‰) and the benzoic acid sample investigated here (−27.77‰) differ by only ∼1.04‰, the accuracy assessment presented herein should be interpreted as a local comparison around the isotopic composition of the reference rather than as validation of linearity across the VPDB scale. Future studies should include at least two isotopically distinct reference materials to establish the slope and intercept of the Orbitrap isotope-response function.

## 4 Conclusions

Across the three injection strategies evaluated, sample introduction mode emerged as the primary factor governing δ^13^C accuracy and precision in Orbitrap-MS measurements of benzoic acid. Dual-inlet delivered the closest overall agreement with the EA-IRMS benchmark, benefiting from continuous reference bracketing that minimizes instrumental drift. Single-inlet maintained competitive within-block precision but proved more susceptible to systematic accuracy offsets, particularly on the Exploris 240. Automated HPLC flow-injection offered the most operationally robust performance, combining accuracy comparable to DI with automation-driven reproducibility and higher sample throughput. Taken together, these results confirm that Orbitrap-MS is a reliable alternative to EA-IRMS for δ^13^C analysis of benzoic acid molecule, and that the choice of injection strategy—rather than instrument generation alone—is the critical variable determining where on the accuracy–throughput continuum a given application will land.

## Supplementary Material



## Notes

Additional ion-source and mass spectrometry parameters for the Q Exactive and Orbitrap Exploris 240 instruments; optimization of automatic gain control settings and benzoic acid concentration; block-level δ^13^C results and associated precision metrics; experimental configurations and replicate results for dual-inlet, single-inlet, and HPLC-based sample introduction; statistical evaluation by ANOVA and Tukey’s test; and detailed equations used to calculate σ_RSE_, σ_PRSE_, shot-noise limit, experimental reproducibility, and δ^13^C values (PDF)
