# Supplementary material for: δ13C Determination of Benzoic Acid by High-Resolution Orbitrap MS: How Sample Introduction Strategy Governs Accuracy and Precision
Source: J Am Soc Mass Spectrom. 2026 Sep 11;37(10):2472–82. doi: 10.1021/jasms.6c00221 (PMC13651617; doi:10.1021/jasms.6c00221)
Supplement: Supplementary file 1 [file js6c00221_si_001.pdf]

Supporting Information

$\delta^{13}C$ Determination of Benzoic Acid by High-Resolution Orbitrap MS: How Sample Introduction Strategy Governs Accuracy and Precision

Giovanni B. Bevilaqua,[a] Hugo G. Machado,[a] Júlio C. O. Ribeiro,[a] Nerilson M. Lima,[b] Alexandre A. Ferreira,[c] Ygor S. Rocha,[c] Gabriel F. dos Santos,[a,]* Boniek Gontijo[a,]*

[a] *Chemistry Institute, Federal University of Goiás, Goiânia, Goiás,74690-900, Brazil*

[b] *UNIFAL, Universidade Federal de Alfenas, Alfenas, 37130-001 Minas Gerais, Brasil*

[c] *PETROBRAS Research and Development Center (CENPES), PETROBRAS, Rio de Janeiro, RJ, 21941-915, Brazil*

Correspondence: Gabriel F. dos Santos (franco.gabriel@ufg.br) and Boniek Gontijo (boniek@ufg.br)

Table of contents:

Table S1: Ion source settings for Orbitrap Q-Exactive and Exploris 240........................3
Table S2: Optimization of automatic gain control (AGC) settings, precision metrics, and scan information using the IAEA-601 reference...3
Table S3: Optimization of benzoic acid concentration: evaluation of ^{13}R stability, within-block standard error, and shot-noise performance 4
Table S4: Block-level $\delta^{13}C$ measurements and associated error components for each injection configuration and instrument...5
Table S5: Results of ANOVA and Tukey tests assessing within-set reproducibility of $\delta^{13}C$ measurements for each injection system and instrument 8

Figure S1: Dual-inlet injection on the QExactive Orbitrap. (Left) Schematic representation of alternating injections of standard (blue) and sample (red) using two syringes. (Center) $\delta^{13}C$ values obtained for benzoic acid replicates. (Right) Mean $\delta^{13}C$ values with standard deviations, referenced against the expected value (−27.77‰)........6
Figure S2: Experimental design and $\delta^{13}C$ results for benzoic acid replicates using the dual inlet configuration on the Orbitrap Exploris 240. (Left) Schematic representation of

alternating injections of sample (red) and standard (blue) blocks. (Center) δ^{13}C values obtained for each replicate, with the dashed red line indicating the expected reference value (−27.77‰). (Right) Mean δ^{13}C values with associated standard deviations, showing high reproducibility across replicates. 6
Figure S3: Single-inlet injection configuration on the QExactive Orbitrap. (Left) Schematic representation of alternating standard (blue) and sample (red) blocks using a single syringe. (Center) δ^{13}C values for benzoic acid replicates, with the dashed red line marking the expected reference value (−27.77‰). (Right) Replicate means ±1σ, showing reproducible results across blocks. 7
Figure S4: Single-inlet injection configuration on the Orbitrap Exploris 240. (Left) Schematic representation of alternating standard (blue) and sample (red) blocks using a single syringe. (Center) δ^{13}C values for benzoic acid replicates, with the dashed red line indicating the expected reference value (−27.77‰). (Right) Replicate means ±1σ, confirming reproducible results across blocks. 7
Figure S5: HPLC-based injection configuration on the Orbitrap Exploris 240. (Left) Schematic representation of alternating standard (blue) and sample (red) blocks delivered via the autosampler loop. (Center) δ^{13}C values for benzoic acid replicates, with the dashed red line marking the expected reference value (−27.77‰). (Right) Replicate means ±1σ, demonstrating the reproducibility of automated injections. 8
Data Processing - Precision and Error Metrics...9

Table S1: Ion source settings for Orbitrap Q-Exactive and Exploris 240

Parameter	Q-Exactive	Exploris 240
Ionization mode	H-ESI (negative)	H-ESI (negative)
Sheath gas (Arb.)	15	1
Aux gas (Arb)	1	1
Sweep gas (Arb)	0	0
Spray voltage	3000V	2700V
Spray current	< 0.3μA	< 0.3μA
Ion Transfer tube temp	320 ºC	320 ºC
Resolution	70000	60000
Scan type	Full scan	Full scan
Polarity	Negative	Negative
Microscans	1	1
Max injection time	50ms	100ms
AGC target	$2x10^5$	$2x10^5$
RS Lens (%)	70%	70%
Mass range (m/z)	120 – 124	120 - 124

Table S2: Optimization of automatic gain control (AGC) settings, precision metrics, and scan information using the IAEA-601 reference

AGC (%)	Replicate	σ_{RSE} (‰)	σ_{SN} (‰)	$\frac{\sigma_{RSE}}{\sigma_{SN}}$	Number of scans	Effective number of ions
5×10^4	1	0.770203	0.559341	1.376983	1152	3.196292e+06
	2	0.775696	0.565219	1.372383	1130	3.130163e+06
	3	0.798600	0.567274	1.407784	1141	3.107519e+06
1.5×10^5	1	0.465571	0.375869	1.238652	1160	7.078254e+06
	2	0.492013	0.379483	1.296533	1137	6.944076e+06
	3	0.475708	0.377135	1.261373	1143	7.030830e+06
2.5×10^5	1	0.386462	0.327819	1.178890	1150	9.305330e+06
	2	0.382642	0.326663	1.171366	1155	9.371320e+06
	3	0.398427	0.332512	1.198232	1116	9.044495e+06

Table S3: Optimization of benzoic acid concentration: evaluation of ^{13}R stability, within-block standard error, and shot-noise performance

	Concentration (μL)	Replicate	^{13}R	σ_{RSE} (‰)	σ_{SN} (‰)	$\frac{\sigma_{RSE}}{\sigma_{SN}}$
Sample	1	1	0.082892	0.73976	0.54088	1.37
		2	0.082905	0.61595	0.47677	1.29
		3	0.082714	0.62706	0.49546	1.27
	5	1	0.082867	0.62155	0.47569	1.31
		2	0.083132	0.67075	0.51785	1.30
		3	0.083161	0.64450	0.51205	1.26
	10	1	0.083305	0.63645	0.49382	1.29
		2	0.083453	0.62928	0.48837	1.29
		3	0.083400	0.61116	0.48564	1.26
	25	1	0.083368	0.63137	0.48635	1.30
		2	0.083331	0.62637	0.48432	1.29
		3	0.083383	0.62479	0.49086	1.27
	35	1	0.083380	0.63423	0.48658	1.30
		2	0.083415	0.63070	0.48555	1.30
		3	0.083469	0.62340	0.48610	1.28
	50	1	0.083427	0.60764	0.48432	1.25
		2	0.083495	0.62776	0.48406	1.30
		3	0.083464	0.62066	0.48289	1.29
Reference	1	1	0.082773	0.65117	0.50151	1.30
		2	0.082851	0.60057	0.47675	1.26
		3	0.082828	0.63253	0.48752	1.30
	5	1	0.083334	0.62252	0.49919	1.25
		2	0.083088	0.65163	0.52512	1.24
		3	0.083558	0.62423	0.48722	1.28
	10	1	0.083591	0.64270	0.49497	1.30
		2	0.083465	0.62373	0.48395	1.29
		3	0.083702	0.59742	0.47887	1.25
	25	1	0.083816	0.62752	0.48797	1.29
		2	0.083539	0.66991	0.53423	1.25
		3	0.083670	0.71149	0.54462	1.31
	35	1	0.083588	0.62572	0.48435	1.29
		2	0.083534	0.71936	0.55625	1.29
		3	0.083608	0.72142	0.54527	1.32
	50	1	0.083911	0.62462	0.49470	1.26
		2	0.083694	0.64216	0.48567	1.32
		3	0.083695	0.61168	0.49064	1.25

Table S4: Block-level δ^{13}C measurements and associated error components for each injection configuration and instrument

Injection System / Equipment	Rep	Sample block	δ^{13}C blocks (‰)	σ_{PRSE} (‰)	σ_{RSE} (‰)	σ_{SN} (‰)	$\frac{\sigma_{RSE}}{\sigma_{SN}}$	δ^{13}C (‰) ± σ_{ER}	Residual vs IRMS (‰)
Dual Inlet - QExactive	1	2	-30.37	0.67	0.61895	0.42853	1.44	-29.31 ± 1.0	-1.54
		4	-27.96	0.60					
		6	-29.59	0.61					
	2	2	-30.44	0.73	0.66848	0.42584	1.57	-30.5 ± 1.43	-2.73
		4	-28.78	0.73					
		6	-32.28	0.83					
	3	2	-26.99	0.65	0.60705	0.42750	1.42	-28.35 ± 1.29	-0.58
		4	-27.97	0.61					
		6	-30.09	0.60					
Dual Inlet - Exploris 240	1	2	-27.23	0.58	0.57388	0.48298	1.19	-28.17 ± 0.67	-0.40
		4	-28.72	0.56					
		6	-28.56	0.57					
	2	2	-26.99	0.61	0.58459	0.48140	1.21	-27.27 ± 0.23	0.50
		4	-27.55	0.57					
		6	-27.28	0.60					
	3	2	-27.46	0.58	0.57373	0.48090	1.19	-27.38 ± 0.41	0.39
		4	-26.84	0.60					
		6	-27.85	0.60					
Single Inlet - QExactive	1	2	-34.78	0.63	0.53417	0.18740	2.85	-30.55 ± 3.12	-2.78
		4	-29.54	0.58					
		6	-27.34	0.65					
	2	2	-27.71	0.52	0.49108	0.19190	2.56	-28.19 ± 0.96	-0.42
		4	-29.53	0.48					
		6	-27.32	0.50					
	3	2	-30.99	0.50	0.48415	0.18997	2.55	-29.72 ± 0.96	-1.95
		4	-28.68	0.54					
		6	-29.48	0.44					
Single Inlet - Exploris 240	1	2	-26.36	0.58	0.52317	0.37618	1.39	-27.04 ± 1.56	0.73
		4	-29.19	0.56					
		6	-25.56	0.58					
	2	2	-30.39	0.57	0.53098	0.37702	1.41	-30.77 ± 0.47	-3.00
		4	-31.43	0.56					
		6	-30.50	0.57					
	3	2	-30.48	0.59	0.54425	0.39033	1.39	-30.39 ± 0.32	-2.62
		4	-29.96	0.57					
		6	-30.73	0.57					
HPLC Injection - Exploris 240	1	2	-27.01	0.42	0.40989	0.32181	1.27	-27.19 ± 0.15	0.58
		4	-27.38	0.41					
		6	-27.18	0.41					
	2	2	-27.53	0.41	0.41321	0.31971	1.29	-28.1 ± 0.4	-0.33
		4	-28.33	0.41					
		6	-28.43	0.41					
	3	2	-27.82	0.42	0.41228	0.31924	1.29	-27.79 ± 0.07	-0.02
		4	-27.86	0.41					
		6	-27.70	0.42					

Reported parameters include the propagated within-block standard error ($\boldsymbol{\sigma_{PRSE}}$), within-block standard error ($\boldsymbol{\sigma_{RSE}}$), shot-noise limit ($\boldsymbol{\sigma_{SN}}$), their ratio ($\boldsymbol{\sigma_{RSE}/\sigma_{SN}}$), and residuals of δ-values relative to the IRMS benchmark (-27.77‰).

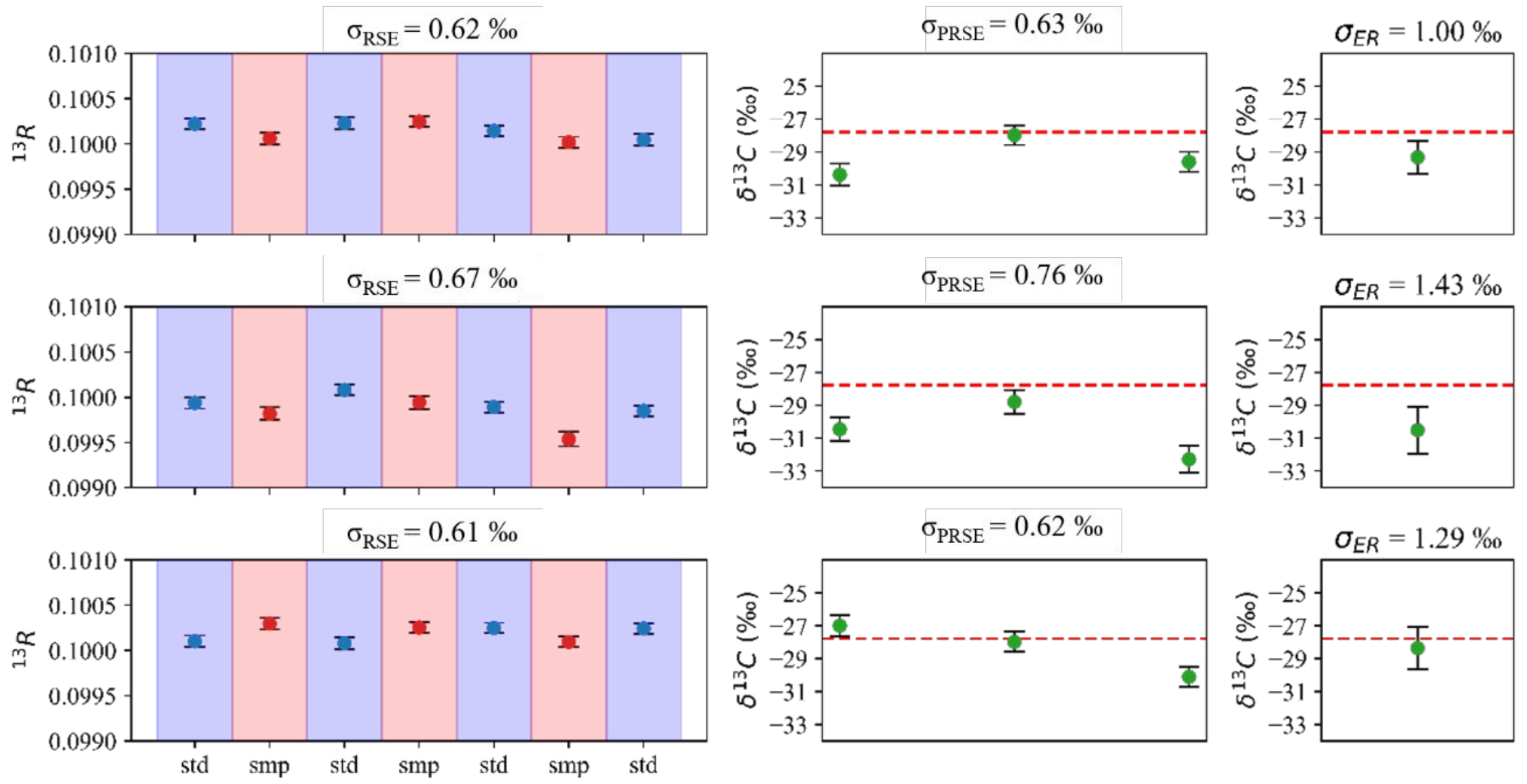

Figure S1: Dual-inlet injection on the QExactive Orbitrap. (Left) Schematic representation of alternating injections of standard (blue) and sample (red) using two syringes. (Center) δ^{13}C values obtained for benzoic acid replicates. (Right) Mean δ^{13}C values with standard deviations, referenced against the expected value (−27.77‰).

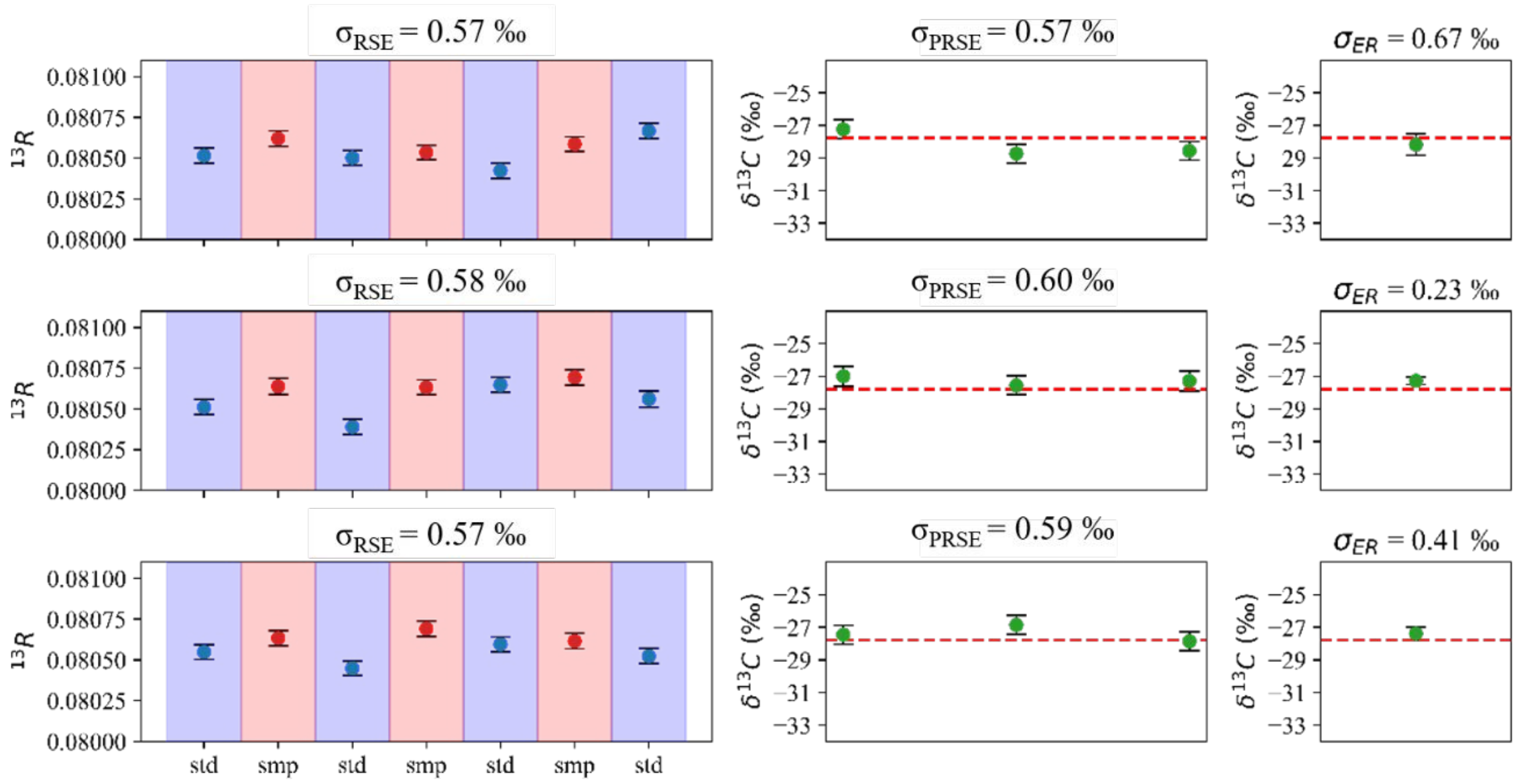

Figure S2: Experimental design and δ^{13}C results for benzoic acid replicates using the dual inlet configuration on the Orbitrap Exploris 240. (Left) Schematic representation of alternating injections of sample (red) and standard (blue) blocks. (Center) δ^{13}C values obtained for each replicate, with the dashed red line indicating the expected reference value (−27.77‰). (Right) Mean δ^{13}C values with associated standard deviations, showing high reproducibility across replicates.

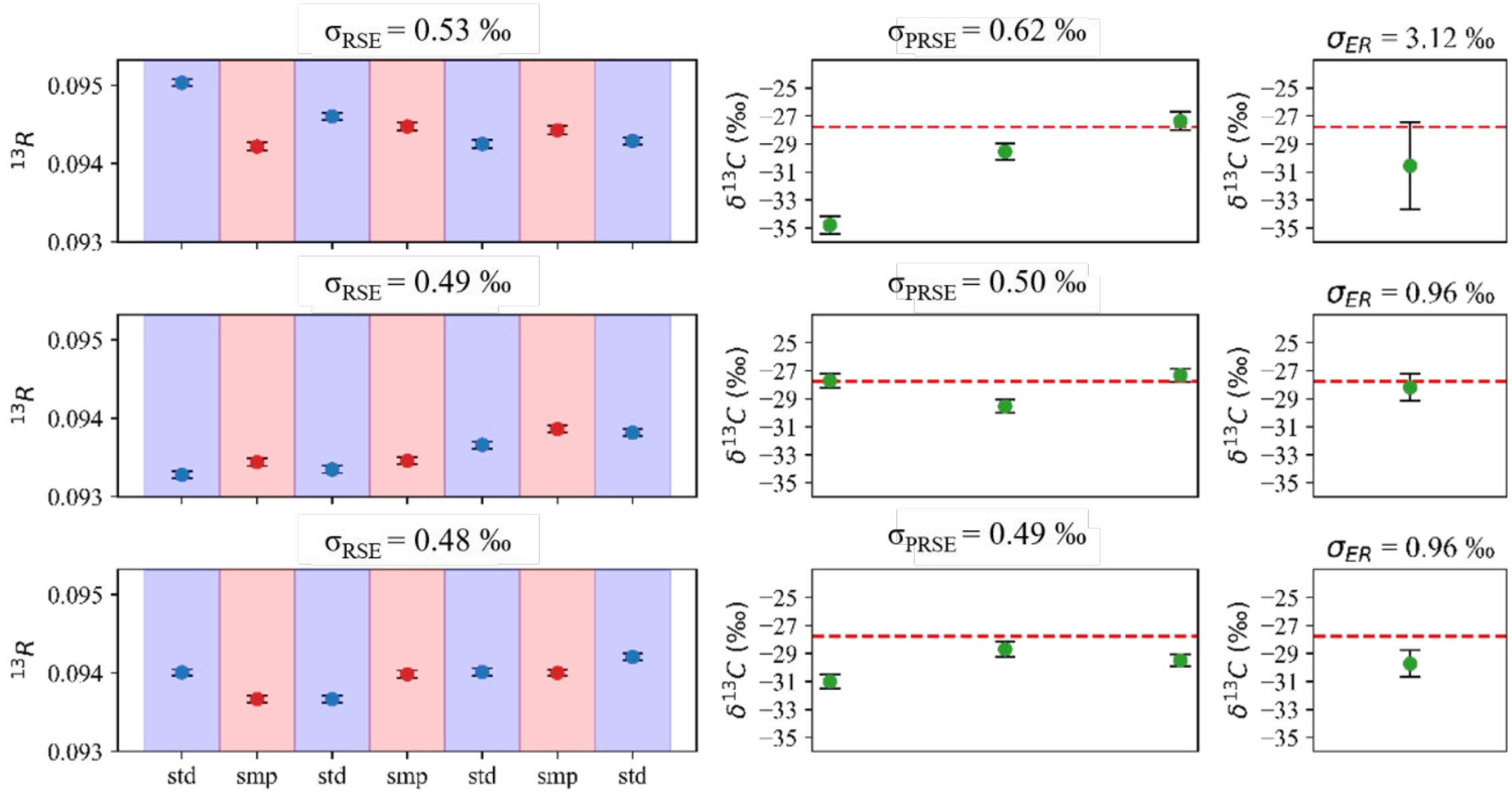

Figure S3: Single-inlet injection configuration on the QExactive Orbitrap. (Left) Schematic representation of alternating standard (blue) and sample (red) blocks using a single syringe. (Center) $\delta^{13}C$ values for benzoic acid replicates, with the dashed red line marking the expected reference value (−27.77‰). (Right) Replicate means ±1σ, showing reproducible results across blocks.

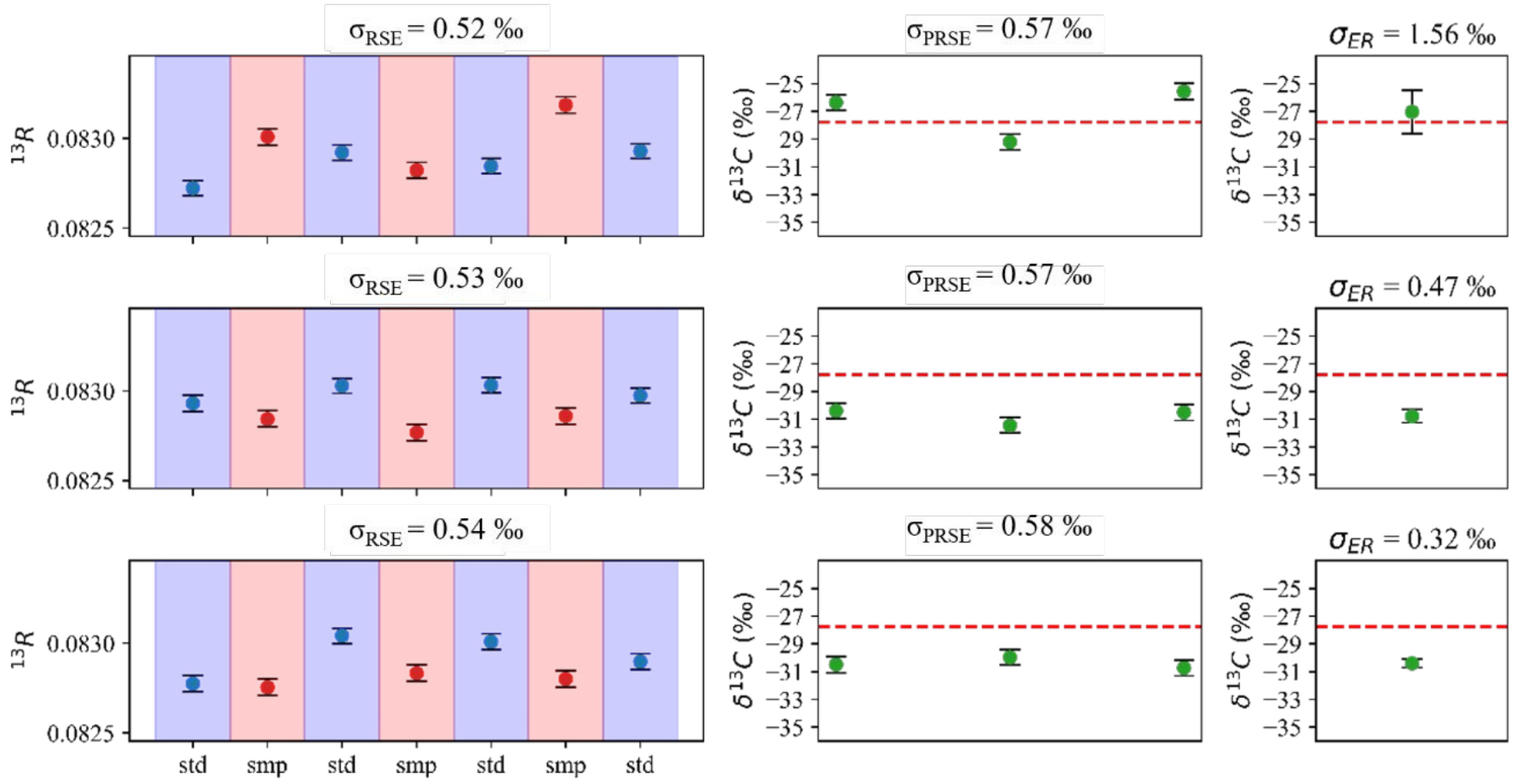

Figure S4: Single-inlet injection configuration on the Orbitrap Exploris 240. (Left) Schematic representation of alternating standard (blue) and sample (red) blocks using a single syringe. (Center) $\delta^{13}C$ values for benzoic acid replicates, with the dashed red line indicating the expected reference value (−27.77‰). (Right) Replicate means ±1σ, confirming reproducible results across blocks.

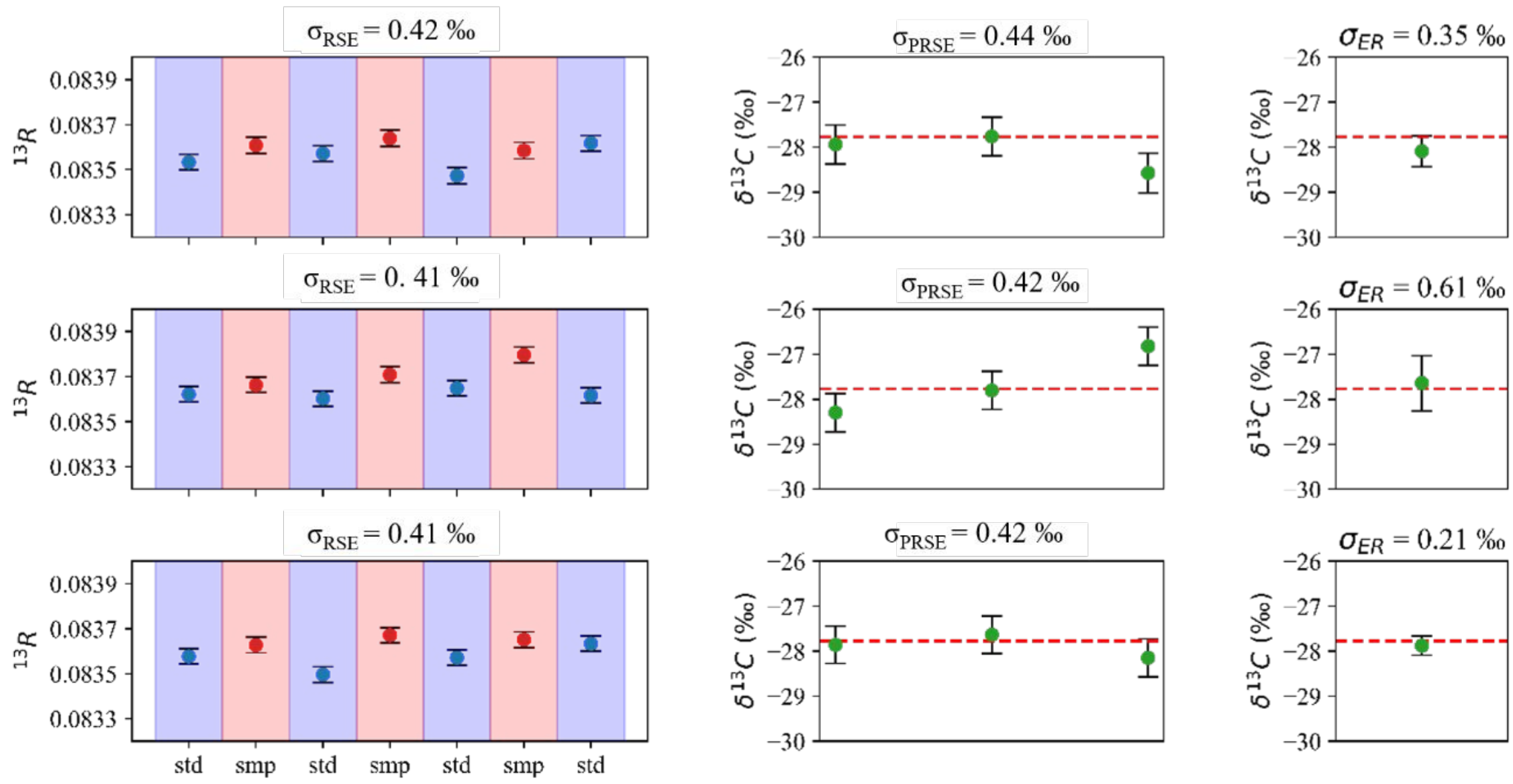

Figure S5: HPLC-based injection configuration on the Orbitrap Exploris 240. (Left) Schematic representation of alternating standard (blue) and sample (red) blocks delivered via the autosampler loop. (Center) $\delta^{13}C$ values for benzoic acid replicates, with the dashed red line marking the expected reference value (−27.77‰). (Right) Replicate means ±1σ, demonstrating the reproducibility of automated injections.

Table S5: Results of ANOVA and Tukey tests assessing within-set reproducibility of $\delta^{13}C$ measurements for each injection system and instrument

**Injection System**	**Equipament**	***p value* (ANOVA)**	**Group 1**	**Group 2**	**Difference between means**	***p value* (Tukey)**	**Rejection**
Dual Inlet	QExactive	0,3313	REP-1	REP-2	-1,14	0,6793	False
			REP-1	REP-3	1,01	0,7351	False
			REP-2	REP-3	2,15	0,3036	False
	Exploris 240	0,1801	REP-1	REP-2	0,92	0,1733	False
			REP-1	REP-3	0,66	0,3563	False
			REP-2	REP-3	-0,26	0,8318	False
Single Inlet	QExactive	0,5528	REP-1	REP-2	2,10	0,5371	False
			REP-1	REP-3	0,67	0,9323	False
			REP-2	REP-3	-1,43	0,7380	False
	Exploris 240	0.0156	REP-1	REP-2	4.27	0.020	True
			REP-1	REP-3	4.28	0.019	True
			REP-2	REP-3	0.02	0.999	False
HPLC Injection	Exploris 240	0.6029	REP-1	REP-2	-0.06	0.900	False
			REP-1	REP-3	-0.24	0.567	False
			REP-2	REP-3	-0.18	0.694	False

Data Processing – Precision and Error Metrics

The *IsotoPy* software provides multiple quantitative indicators of measurement precision, which are computed automatically at different stages of data processing and δ-value calculation. These include the within-block standard error (σ_{RSE}), the propagated within-block standard error (σ_{PRSE}), and the experimental reproducibility error (σ_{ER}). Together, these metrics describe the short-term stability, propagated uncertainty, and overall reproducibility of the Orbitrap-based isotope-ratio measurements.

Within-block standard Error (σ_{RSE})

The within-block standard error (σ_{RSE}) represents the precision within a single acquisition block and corresponds to the relative standard error (RSE) of the isotopic ratios, expressed in per mil (‰). It is calculated as:

$$\sigma_{RSE} = \frac{SEM}{\bar{R}} \times 1000 \tag{S1}$$

where SEM is the standard error of the mean of the isotopic ratios and $\bar{R}$ is their average. This parameter quantifies the scan-to-scan variability of isotopic ratios within each block and therefore reflects short-term instrumental stability. For multi-block acquisitions, the reported σ_{RSE} corresponds to the mean of all block-level σ_{RSE} values.

Shot-Noise Limit (σ_{SN})

In any isotopic measurement, there exists a fundamental precision limit imposed by counting statistics, known as the shot-noise limit (σ_{SN}). This limit is the theoretical best precision achievable for an isotopic ratio given the finite number of ions counted for each isotopologue. In essence, it defines the smallest possible σ_{RSE} attainable if stochastic (Poisson) ion-counting noise were the only source of error. The σ_{SN} for a ratio can be estimated from the total ion counts of the two relevant ion beams (e.g., M0 and M+1) as:

$$\sigma_{SN} = \sqrt{\frac{1}{\sum IonCounts_{M0}} + \frac{1}{\sum IonCounts_{M+1}}} \tag{S2}$$

where $\sum IonCounts_{M0}$ and $\sum IonCounts_{M+1}$ denote the summed counts of the M0 and M+1 ion beams, respectively. The σ_{SN} value provides a benchmark for the minimum RSE expected from counting statistics alone. Conversely, if the actual σ_{RSE} is significantly higher than the σ_{SN}, it suggests additional sources of variability affecting the measurement.

In practice, the $\frac{\sigma_{RSE}}{\sigma_{SN}}$ ratio is used as a data-quality indicator: values of this ratio near 1 imply that the measurement approaches the theoretical noise floor (i.e., optimal precision), whereas values substantially greater than 1 indicate degraded precision. Previous studies have shown that when $\frac{\sigma_{RSE}}{\sigma_{SN}}$ exceeds approximately 2 (i.e., the acquisition error is about twice the shot-noise limit), the corresponding data should be considered low-quality and rejected[1].

Standardization and Delta Calculation

When the standardization protocol is applied for δ-value determination, a linear regression is constructed using the isotope ratio of the reference blocks (1, 3, 5, and 7) to predict the expected ratio at each sample block (2, 4, and 6). In this case, the propagated within-block standard error (σ_{PRSE}) combines the within-block standard error with the prediction uncertainty derived from the regression model, according to:

$$\sigma_{PRSE} = \sqrt{\sigma^2{}_{RSE} + \sigma^2{}_{pred}} \quad \text{(S3)}$$

The regression prediction uncertainty ($\sigma^2{}_{pred}$) is calculated as:

$$\sigma_{pred} = \sqrt{\frac{\sum\left(R_{smp}^{real} - R_{smp}^{pred}\right)^2}{n-2}} \times \sqrt{\frac{1}{n} + \frac{\left(x_{smp} - \bar{x}_{ref}\right)^2}{\sum\left(x_{ref} - \bar{x}_{ref}\right)^2}} \quad \text{(S4)}$$

where R_{smp}^{real} and R_{smp}^{pred} are the measured and predicted isotopic ratios, respectively; x_{ref} and x_{smp} are the acquisition times of the reference and sample blocks; and n is the number of reference points (typically four).

The experimental reproducibility error σ_{ER} represents the variability among the three $\delta^{13}C$ values obtained within a single seven-block acquisition, reflecting the overall reproducibility of the method under identical analytical conditions:

$$\sigma_{ER} = stdev(\delta_1,\delta_2,\delta_3) \quad \text{(S5)}$$

σ_{ER} integrates the cumulative effects of acquisition precision, instrumental drift, and data-processing variability. It thus serves as a direct indicator of run-to-run repeatability and the robustness of the isotope-ratio determination protocol.

In this study, the $\delta^{13}C$ values were referenced to the VPDB scale using the certified value of the benzoic acid standard IAEA-601 ($\delta^{13}C_{STD/VPDB}$=-28.81 ‰). The unlabeled benzoic acid analyzed in this work exhibited a $\delta^{13}C$ value of -27.77 ‰, determined independently by GC-IRMS, and this value was adopted as the accuracy benchmark for evaluating $\delta^{13}C_{sample/VPDB}$ measurements. Accordingly, accuracy was assessed by comparing measured $\delta^{13}C_{STD/VPDB}$ values against this reference, while σ_{RSE}, σ_{PRSE}, and σ_{ER} were used to characterize precision and reproducibility.
